# Hippocampal Glial Alterations Are Associated with Mitochondrial Changes and Reduced Glucocorticoid Receptor Density in Suicides

**DOI:** 10.3390/ijms27219700

**Published:** 2026-10-29

**Authors:** Andrea Judith Vázquez-Hernández, Yali Escribano-Cadena, Julio César Morales-Medina, Amira Flores, Laura Alejandra Mendoza-Larios, Roberto Cuauhtémoc Mendoza-Morales, Humberto Nicolini, Hiram Tendilla-Beltrán, Fernando García-Dolores, Gonzalo Flores

**Affiliations:** 1 Instituto de Fisiología, Benemérita Universidad Autónoma de Puebla (BUAP), Puebla 72570, Mexico; andrea-vh95@hotmail.com (A.J.V.-H.); yaliescribano@yahoo.com (Y.E.-C.); amrayo@yahoo.com.mx (A.F.); 2 Centro de Investigación en Reproducción Animal, Cinvestav-Universidad Autónoma de Tlaxcala, Tlaxcala 90120, Mexico; jmoralesm@cinvestav.mx (J.C.M.-M.); hiram.tendilla@cinvestav.mx (H.T.-B.); 3 Instituto de Ciencias Forenses (INCIFO), Tribunal Superior de Justicia de la Ciudad de México (TSJCDMX), Mexico City 06720, Mexico; laura.larios@tsjcdmx.gob.mx (L.A.M.-L.); robertomendozamorales@hotmail.com (R.C.M.-M.); 4 Instituto Nacional de Medicina Genómica (INMEGEN), Mexico City 14610, Mexico; hnicolini@inmegen.gob.mx; 5 Hospital Psiquiátrico Dr. Rafael Serrano “El Batán”, Puebla 72960, Mexico

**Keywords:** inflammation, neuronal density, oxidative/nitrosative stress

## Abstract

Suicide is a major public health concern frequently associated with depressive disorders and neurobiological alterations within the hippocampus. This study investigated biomarkers related to neuroinflammation, oxidative/nitrosative stress, and mitochondrial labeling in the CA1 region of the hippocampus of young and adult individuals of Mexican ancestry who died by suicide. Postmortem hippocampal CA1 samples were obtained from young and adult suicide cases and age-matched controls. Cellular markers of microglia (ionized calcium-binding adapter molecule 1, Iba1) and astrocytes (glial fibrillary acidic protein, GFAP), neuronal density, glucocorticoid receptor expression, and mitochondrial labeling were evaluated and compared among groups. Suicide cases exhibited increased microglial and astrocyte cell density indicated by increased Iba1 and GFAP immunoreactivity in both age groups. In contrast, mitochondrial labeling and glucocorticoid receptor immunoreactivity were reduced in suicide cases, regardless of age. Additionally, a significant reduction in neuronal density was detected exclusively in young individuals who died by suicide. Together, these findings indicate age-independent alterations in glial immunoreactivity, mitochondrial labeling, and glucocorticoid receptor expression, alongside reduced neuronal density in the hippocampal CA1 region of younger suicide cases, supporting the involvement of neuroinflammation-related processes in suicide. Despite the limited sample size, these findings provide insight into potential neurobiological mechanisms underlying suicide and may contribute to the identification of biomarkers and therapeutic targets for individuals at risk.

## 1. Introduction

Suicide is one of the most complex human behaviors and has increased worldwide in recent years. Although females experience suicidal thoughts and attempts more frequently, males are more likely to die by suicide [1,2]. Furthermore, the number of adolescents and young adults (14 to 28 years old) who have died by suicide has increased significantly over the past 50 years [1,3]. In fact, suicide is the second leading cause of death among young people in some countries [3,4]. Several psychiatric disorders have been associated with suicidal behavior, including major depressive disorder, schizophrenia, bipolar disorder, as well as substance abuse [5,6,7]. However, suicide, particularly among young people, may also be associated with other factors, including childhood abuse, prenatal stress, poor maternal care, etc. Thus, multiple biological and environmental factors may contribute to suicidal behavior [8].

Although numerous studies have investigated suicide among adolescents and young adults, partly because of its high prevalence in these age groups compared with older individuals [1,3], whether and how the neurobiological alterations associated with suicide differ across age groups remains insufficiently understood [9]. Comparing these age groups may help determine whether these alterations are shared across ages or exhibit age-related differences [10]. This comparison is particularly relevant for understanding the potential contribution of age to the neurobiological alterations associated with suicide [11].

Regarding the brain, the limbic system is known to be involved in suicidal behavior. This system consists of a set of closely connected structures that participate in the regulation of emotions [12]. Among the most crucial structures are the prefrontal cortex (PFC), hippocampus, amygdala, hypothalamus, nucleus accumbens (NAcc), and limbic thalamus. These areas are associated with glutamatergic excitatory connections between the PFC, hippocampus, basolateral amygdala (BLA), and the dorsomedial (DM) nucleus of the thalamus. Furthermore, these nuclei send glutamatergic projections to the NAcc, also known as the reward nucleus, and to the hypothalamus, critical in the control of the hypothalamic–pituitary–adrenal (HPA) axis. This axis is related to stress by means of the release of cortisol [13]. Consequently, these regions participate in both the regulation of emotional processes, ranging from reward to pleasure/displeasure, and the hormonal control of the stress response. Indeed, the role of these connections is critical in learning, regulating, and maintaining emotions in the face of life’s eventualities and their relationship with stress [14].

While the hippocampus, as part of the limbic system, has been widely studied in multiple mental disorders [15], including suicide [16], there is still scarce information regarding subfield-specific hippocampal alterations in suicide. The hippocampus is made up of three regions: the hippocampal formation, the dentate gyrus, and the subiculum. The hippocampal formation is made up of areas of the corpus ammonis (CA), also known as Ammon’s Horn, described as CA1, CA2, CA3, and CA4, which were first described by Lorente de No [17]. Information is intrinsically processed by an excitatory glutamatergic tri-synaptic circuit between the spheroidal neurons of the dentate gyrus, which sends projections to the pyramidal neurons of the CA3 region through the mossy fibers, and the pyramidal neurons of the CA3 region send connections to the pyramidal neurons of the CA1 region through Shafer collaterals [18]. Consequently, information from the hippocampus goes out through the pyramidal neurons of the CA1 region to these regions [19].

Interestingly, changes in oxidative/nitrosative stress, major histocompatibility complex class II (MHCII), mitochondrial function, glucocorticoid receptors and some inflammatory markers have been reported in the PFC of individuals who died by suicide [20,21,22]. To date, it is unknown whether these processes are present in the hippocampi of individuals who died by suicide, and even less is known about whether they differ between age groups.

In the present study, we evaluated inflammatory markers such as glial fibrillary acidic protein (GFAP), to identify astrocytes, and the ionized calcium-binding adapter molecule 1 (Iba1), which is a microglia-specific protein. In addition, stress was analyzed through glucocorticoid receptors, mitochondrial labeling was assessed using the MitoTracker test, and neuronal counts were measured using stereology. These analyses were performed in the CA1 region of the dorsal hippocampus of individuals who died by suicide and their non-suicidal controls, highlighting two ages, young and adult.

## 2. Results

### 2.1. Descriptive Statistics of the Included Samples

The sociodemographic and clinical characteristics of the study cohort are summarized in Table 1. The study included 26 postmortem subjects distributed among four groups: Young Suicide (*n* = 8), Adult Suicide (*n* = 7), Young Control (*n* = 6), and Adult Control (*n* = 5). Mean ages were 22.5 years (range 18–28), 46.5 years (range 31–61), 21.2 years (range 18–25), and 41 years (range 34–45), respectively. The male-to-female ratios were 5:3 in the Young Suicide group, 6:1 in the Adult Suicide group, 4:2 in the Young Control group, and 5:0 in the Adult Control group. Suicide cases died by hanging, whereas controls died from various non-suicidal causes. The prevalence of documented depression was higher in Adult Suicide cases (66.7%) than in the other groups. Regarding tissue laterality, right hemispheres predominated in the Young Suicide (62.5%) and Adult Suicide (66.7%) groups, whereas the Young Control group was evenly distributed between hemispheres. In contrast, all Adult Control samples were obtained from the left hemisphere. Notably, all subjects in the Adult Control group were male. This unequal distribution of hemisphere selection, together with the sex imbalance across groups, represents a potential limitation of the study. Although the impact of hemispheric lateralization on the molecular endpoints evaluated remains uncertain, hemisphere-specific differences in gene expression, cellular composition, or neurobiological organization cannot be completely excluded and should therefore be considered when interpreting the findings.

### 2.2. Increased Glial Cell Density in the CA1 Region of Suicides

The density of two types of glial cells were evaluated in the present study: astrocytes and microglia marked with GFAP and Iba1, respectively (Figure 1(Ai,Bi)). The number of GFAP-positive astrocytes was increased (two-way ANOVA, death by suicide F (1, 16) = 54.7, *p* < 0.0001, η^2^ = 0.77; age, F (1, 16) = 16, *p* < 0.001, η^2^ = 0.50), as were Iba1-positive microglia (two-way ANOVA, death by suicide F (1, 16) = 67.68, *p* < 0.0001, η^2^ = 0.81; age, F (1, 16) = 13.7, *p* < 0.001, η^2^ = 0.46). Post hoc analysis revealed that individuals of all ages who die by suicide show an increase in the number of GFAP-positive astrocytes in the CA1 region of the hippocampus (Young *p* = 0.002, adult, *p* < 0.0001) (Figure 1(Aii)). Similarly, individuals who die by suicide also exhibit an increase in the number of Iba1-positive microglia in the CA1 region of the hippocampus (young *p* < 0.0001, adult, *p* < 0.001); there was also an increase in the number of Iba1-positive microglia in Adult Controls compared to Young Controls (*p* = 0.03) (Figure 1(Bii)).

### 2.3. Decreased Glucocorticoid Receptor-Reactive Cells in the CA1 Region of Suicides

Glucocorticoid receptors are involved in the functionality of the HPA axis. It has been suggested that this area is dysfunctional in depression. Therefore, we evaluated the density of glucocorticoid receptors (two-way ANOVA, death by suicide F (1, 16) = 78.12, *p* < 0.0001, η^2^ = 0.83; Figure 2(Ai)). Statistical analysis reveals that individuals who die by suicide show a reduction in the number of glucocorticoid receptor-reactive cells (*p* < 0.0001) in the CA1 region of the hippocampus (Figure 2(Aii)).

### 2.4. Decreased MitoTracker-Positive Cell Density in the CA1 Region of Suicides

Statistical analysis of the mitochondrial labeling by MitoTracker-positive cells (two-way ANOVA, death by suicide F (1, 16) = 44.75, *p* < 0.0001, η^2^ = 0.74; Figure 2(Bi)) reveals that individuals who die by suicide have a reduced number of reactive cells (young *p* < 0.01, adults *p* < 0.001) in the CA1 region of the hippocampus, with no differences between groups of the same condition (see Figure 2(Bii)).

### 2.5. Age-Dependent Reduction in Hippocampal CA1 Neuron Number in Suicides

Statistical analysis of neuron count in the CA1 region of the hippocampus was performed by stereology (Figure 3A). One case was removed due to its Gundersen Coefficient > 0.1 (Table 1). The results reveal that young individuals who died by suicide have a reduced neuronal count (two-way ANOVA, death by suicide F (1, 20) = 5.3, *p* = 0.0316, η^2^ = 0.21) compared to the Young Control group, but these differences do not extend into adulthood (Figure 3B).

## 3. Discussion

The main objective of this study was to analyze biomarkers associated with glial density, oxidative/nitrosative stress, and mitochondrial labeling in the CA1 region of the hippocampus of young and adult individuals who died by suicide. We found that both Young and Adult Suicide groups exhibited increased numbers of Iba1-positive microglia and GFAP-positive astrocytes in the hippocampus. Furthermore, these individuals exhibit decreased hippocampal mitochondrial labeling and glucocorticoid receptor density, regardless of age. Interestingly, the reduction in hippocampal neuronal density observed in young individuals who died by suicide is not extended to the adult group.

Iba1 is a protein typically localized to microglia and macrophages [23]. As such, its increased density may suggests glial proliferation and may be considered a marker of inflammation [24]. Interestingly, our results suggest that regardless of age, individuals who have died by suicide show an increase in this biomarker, suggesting the presence of an inflammatory process in the CA1 region of the hippocampus in these individuals. The role of microglia in the hippocampus is quite complex. Under physiological conditions, they can contribute to proper functionality of synaptic communication by promoting the removal of waste products, such as dysfunctional synapses. However, under pathological conditions, microglia can shift from being protective to toxic by promoting the release of proinflammatory cytokines, some of which have neurotoxic properties [25,26]. All of this can lead to impairments in synaptic communication and neuroplasticity through damage to neuronal integrity, as suggested by our stereological results, which show a reduction in neuronal density in young individuals who died by suicide.

Increased GFAP expression and immunoreactivity are commonly used as indicators of astrogliosis, a process associated with inflammatory conditions in the brain [27,28]. GFAP is a major component of the astrocytic cytoskeleton, and astrocytes play important roles in regulating synaptic plasticity and neuronal communication under physiological conditions [29]. Under pathological conditions, however, increased GFAP immunoreactivity may reflect astrocyte reactivity and potentially disrupt neuronal communication. In the hippocampus, such alterations may affect neural processes involved in learning and, particularly, memory [29,30].

Interestingly, and consistent with our findings, a case report described increased GFAP levels in the hippocampus of a patient who died by suicide [31]. Other studies have reported alterations in glial markers in subjects with depression. One study found increased glial density in the hippocampus of subjects with depression [32], whereas another found no difference in the density of GFAP-positive cells in the CA1 region of the hippocampus in subjects diagnosed with depression. However, the latter study reported a positive correlation between GFAP-positive cell density and age in depressed subjects, an association that was not observed in controls [33]. These findings should be interpreted cautiously in the context of the present study, as they were reported in subjects with depression, including individuals who died by suicide, whereas depression was not the predominant etiology in our cohort. Nevertheless, these observations are consistent with our working hypothesis that increased levels of the inflammatory cell markers Iba1 and GFAP may indicate an inflammatory process in the CA1 region of the hippocampus in individuals who died by suicide.

Given that the neuroinflammatory hypothesis of suicide is a key framework for interpreting the present findings [11], it is important to consider the multiple mechanisms involving Iba-1-positive and GFAP-positive cells. Astrocytes contribute to blood–brain barrier (BBB) integrity, and proinflammatory astrocytic phenotypes may compromise BBB function. Recent studies have also suggested impaired BBB integrity and function in suicide [34,35]. In turn, increased BBB permeability may facilitate the infiltration of peripheral immune cells, including myeloid cells such as macrophages. Therefore, increased Iba-1 immunoreactivity may not be attributable solely to increased microglial cell density.

Our study observed a reduction in glucocorticoid receptors in the CA1 of the hippocampus. In agreement with our results, some patients who died by suicide show a reduction in the expression of these receptors in the hippocampus, both overall and in young people [36,37,38]. The suggested mechanism is that the hippocampus exerts inhibitory control over the HPA axis through glucocorticoid receptor-mediated negative feedback. When there is a decrease in this negative feedback (reduced glucocorticoid receptor expression or sensitivity), as a result, there is sustained or excessive release of adrenocorticotropic hormone (ACTH), corticotropin-releasing hormone (CRH) and cortisol [39] in patients with depression or those with chronic stress. In turn, the overstimulation of cortisol receptors causes a down-regulation phenomenon, which results in a reduction in their expression. Several findings suggest this, such as the dexamethasone suppression test [40]. Furthermore, recent work from our group has shown that the adenohypophysis is enlarged in young individuals who died by suicide [41] and that the prevalence of microadenomas is increased [42].

Interestingly, consistent with our findings, reduced mitochondrial MitoTracker labeling was observed in those who died by suicide, which may reflect alterations in mitochondrial abundance or function (for details, see the Section 4.2). Several recent reports have shown that mitochondrial function is altered in patients with depression [43,44]. Mitophagy dysfunction has even been reported in individuals with depression or chronic stress [44,45]. Mitophagy implies that neurons remove damaged and aged mitochondria in response to stress [44,46]. Furthermore, recent reports at the level of the dorsolateral PFC of patients who died by suicide suggest that the analysis of the transcriptome of these patients finds alterations in mitochondrial function. On one hand, an increase is found in the expression of genes with mitochondrial function and related to oxidative phosphorylation [22]. The increased expression of genes related to mitochondrial function may be caused by a response related to the decrease in reduced function, as suggested by our results in the hippocampus, due to dysfunctional mitophagy. A hypothetical mechanism integrating the findings of this study and the potential interaction among these processes is presented in Figure 4.

Finally, one of our most relevant findings of this study is the age-dependent pattern observed in hippocampal neuronal density. While no significant differences were detected between adults who died by suicide, younger individuals exhibited a marked reduction in hippocampal neuronal density. In addition, the adult groups show a reduction compared to the Young Control group, suggesting that this is due to changes associated with aging [47]. Furthermore, the reduction in neuronal density in the CA1 region of the hippocampus at an early age could have serious implications, as this region is the output of tri-synaptic communication (DG-CA3-CA1) with projections to the PFC, the BLA, and the hypothalamus. It has been suggested that the projection to the hypothalamus plays a role in the regulation of the HPA axis, with consequences when there is axis dysregulation, as has been suggested in depression and in individuals who have died by suicide [41,42,44,48].

What other mechanisms could reduce the neuronal population in the CA1 region? Recent reports suggest that neuronal autophagy is increased in patients with depression or chronic stress [49]. Interestingly, a recent report suggests that there is an increase in the expression of autophagy-related genes in individuals suffering from depression [49,50]. Decreased mRNA expression of AKT1 and MTOR have also been reported in the PFC of patients with bipolar disorder. In addition to postmortem studies of depressed patients, it has been reported that mTOR signaling is reported to be compromised at the PFC level. These changes may favor the induction of neuronal autophagy. Consequently, the reduction in the neuronal population in young people who died by suicide could be due to neuronal autophagy; however, further studies analyzing markers for this function are needed.

A limitation of this study is the relatively small sample size, which reflects the inherent difficulty in obtaining well-characterized postmortem hippocampal tissue from individuals who died by suicide. Nevertheless, given the clinical and public health significance of suicide, particularly among young individuals, we believe that these findings provide valuable insights into the neurobiological alterations associated with suicidal behavior. Another limitation is the unequal distribution of hemispheric samples across groups. Specifically, all Adult Control samples were derived from the left hemisphere, whereas the suicide groups included samples from both hemispheres. Furthermore, the Adult Control group consisted exclusively of male subjects. These factors may introduce potential confounding effects related to hemispheric lateralization and sex-specific biological differences. An additional limitation is that all suicide cases involved death by hanging, whereas the control groups included subjects with heterogeneous causes of death. Hanging is the most prevalent suicide method in Mexico City, supporting its epidemiological relevance in this context [1,51]. Furthermore, our research group has published four reports investigating the genetics and neurobiology of suicide in individuals who died by hanging [41,42,52,53]. However, hanging can rapidly compromise cerebral blood flow and oxygenation, potentially affecting neuroinflammatory and mitochondrial markers. Therefore, despite the greater homogeneity of the suicide group in terms of the method of death and the relevance of this approach to our previous research, the potential contribution of hanging-related hypoxia cannot be completely ruled out [54,55]. Finally, the lack of data on exposure to psychotropic drugs (e.g., antidepressants and antipsychotics) is another limitation of this study, as both preclinical and clinical evidence indicates that these drugs can modulate neuroinflammation and glial phenotype [56,57], as well as glucocorticoid receptor expression and activity [58,59].

Therefore, the findings should be interpreted with caution and warrant replication in larger cohorts with more balanced demographics, psychotropic medication use, and anatomical representation. The availability of more data on sample variables, such as PMI, tissue pH, agonal state and fixative/paraffin periods, and the inclusion of non-suicidal individuals who died from asphyxia would contribute to clarify the relative contribution of suicidal pathology versus hypoxic injury.

## 4. Materials and Methods

All postmortem tissue samples included in this study from the CA1 region of the human hippocampus were obtained from the Institute of Forensic Sciences (INCIFO) in Mexico City and are part of the Tissue Collection of this Institute for research projects. The study included postmortem samples from 26 subjects distributed across two main categories, Control and Suicide, as well as two age cohorts: Young (18–29 years old) and Adult (31–61 years old). This resulted in four experimental groups: Young Suicide (*n* = 8), Adult Suicide (*n* = 7), Young Control (*n* = 6), and Adult Control (*n* = 5). All the samples have a postmortem interval from 12 to 15 h, and the demographic and clinical characteristics, including age, sex, psychiatric history, and tissue laterality, are summarized in Table 1. Additionally, inclusion/exclusion criteria are detailed in the Supplementary Material.

For sample analysis and evaluation, a unique, anonymous code was assigned to each case, and the evaluation was double-blinded, with the analyst unaware of the sample characteristics. The protocol was approved by the Research Ethics Committee of the Institute of Forensic Sciences (INCIFO) of the TSJCDMX (09-CEI-022-2016082-015-2022; approved on 8 September 2022) and was carried out in accordance with the National Guide for the Execution of Research Projects in Human Health (Official Mexican Standard NOM-012-SSA3-2012) and the international standards for research in human subjects included in the 1975 Declaration of Helsinki.

The method pipelines are schematically represented in Figure 5. The samples were approximately 2 cm^3^ blocks immersed in 4% paraformaldehyde. Upon receipt, they were marked with a code for double-blind processing, as described above. They were immediately embedded in paraffin using a tissue processor (Leica TP10120) and were subsequently cut using a rotatory microtome (Bright type 5040) at 10 µm for immunohistochemistry and at 30 µm for stereological studies. The slices were then mounted on slides previously prepared with poly-l-lysine.

All the samples were employed for stereological cell count assessments, but for immunofluorescence assays, in groups with more than 5 cases, the n was adjusted to 5. Details are shown in Table 1.

### 4.1. Immunofluorescence

The procedure was based on the methodology described by García-Dolores et al., 2025 [60], with some modifications. Briefly, the CA1 sections were first deparaffinized using a double boiler and by two 10 min immersions in xylene, followed by an incubation in a 1:1 xylene–ethanol mixture, then by two immersions in 100% ethanol (the first for 10 min and the second for 3 min). The tissues were immediately rehydrated in decreasing concentrations of ethanol (96%, 80%, 70%) for 10 min each. Finally, they were washed in distilled water for 5 min.

Once hydrated, the CA1 sections were rinsed twice with 0.1 M phosphate-buffered saline (PBS pH 7.4) for 5 min. The next step was antigen retrieval, which was achieved by heating the tissue samples in citrate buffer (10 mM, pH 6) for 30 min. Tissues were then allowed to cool to room temperature for 20 min. Afterward, two washes were performed with 0.1 M PBS for 5 min each.

To increase cell permeability, tissue was washed with 0.2% Triton X-100 for 20 min. This was followed by two washes with 0.1 M PBS for 5 min each. Then, the tissue was immersed in 1x Tris-EDTA for 30 min, followed by two 0.1 M PBS washes for 5 min each. The next step was to block nonspecific sites. For this purpose, the tissue was incubated at room temperature in a 2% IgG-free bovine serum albumin (BSA) solution diluted in 0.1 M PBS for 2 h. After this period, the tissue was washed twice with 0.1 M PBS for 5 min each. Next, the tissue was incubated with the primary antibody diluted in 0.1M PBS for 24 h at 4 °C. Primary antibodies were: Iba1 (Cell Signaling, 17198S, dilution 1:200; Danvers, MA, USA) and GFAP (Cell Signaling, 3670, dilution 1:600; Danvers, MA, USA). This was achieved in a closed, humid chamber in the dark. Subsequently, the tissue was washed twice with 0.1 M PBS for 5 min each. Then, the secondary antibody was diluted in 0.1 M PBS with 1% IgG-free BSA and incubated for 2 h in the dark, followed by two 0.1 M PBS washes performed for 5 min each. The secondary antibodies used were: Rhodamine (Jackson Immuno Research Laboratories Icn. West Grove, PA, USA, red channel, 711-295-152, dilution 1:200) and Alexa Fluor^®^ 488 (Cell Signaling, green channel, 4408, dilution 1:1000; Danvers, MA, USA). For glucocorticoid receptor detection, anti-glucocorticoid receptor coupled to Alexa Fluor^®^ 488 was used (Cell Signaling, 12007, dilution 1:600; Danvers, MA, USA), incubated 24 h at 4 °C. Finally, the coverslip was secured with 20 μL of VectaShield mounting medium with DAPI (Vector Laboratories, H-1200-10; Newark, CA, USA). The mount was then sealed using transparent varnish around the coverslip.

### 4.2. Mitochondrial Labeling

For the slides used in the mitochondrial labeling protocol, the MitoTracker Red CMXROS kit (Thermo Fisher Scientific; Waltham, MA, USA) was used to assess the density of MitoTracker-positive cells; that is, cells exhibiting detectable mitochondrial membrane potential. The procedure was based on the methodology described by García-Dolores et al., [60] with some modifications. Briefly, after the deparaffinization procedure and the two 0.1 M PBS washes, 0.1 M PBS containing 0.2% Triton X-100 solution was applied for 20 min. Then, two 0.1 M PBS washes were performed for 5 min each. One-time Tris-EDTA was applied for 20 min, and two more 0.1 M PBS rinses were performed for 5 min each. Afterwards, antigen retrieval was performed with 1 X SSC buffer (pH 7). The slides were immersed in the buffer at approximately 70 °C for 30 min. The solution was then allowed to cool to room temperature for 30 min. Two washes with 0.1 M PBS were performed for 5 min each. Next, 2% BSA solution was applied for 1 h and then rinsed twice with 0.1 M PBS for 5 min each. Finally, samples were incubated in MitoTracker working solution (150 nM) at a cold temperature for 1 h. Samples were then washed twice with PBS for 5 min each, slides were cover-slipped using 20 μL of VectaShield mounting medium with DAPI (Vector Laboratories, H-1200-10; Newark, CA, USA) per tissue. Finally, clear varnish was applied to the edges to secure the coverslip.

### 4.3. Microscopic Analysis

Photomicrographs were taken using a fluorescence microscope (Leica DMLS with fluorescence). Images were obtained with a digital camera (Leica microsystems; Wetzlar, Germany), coupled to a Leica DMLS microscope, and analysis was performed using Image J (version 1.53; National Institutes of Health, Bethesda, MD, USA). Three consecutive sections of the CA1 region of the hippocampus were analyzed at 40× magnification with the appropriate filter for each marker. In each slice, four fields corresponding to the CA1 region of the hippocampus were examined. Cells immunoreactive to Iba1, GFAP, glucocorticoid receptors and MitoTracker were quantified. Quantification was performed manually. A cellular profile was considered positive when fluorescence corresponding to the marker under evaluation was spatially associated with DAPI-positive nucleus. Image-acquisition settings were established separately and maintained across subjects and experimental groups for each marker. The same camera configuration and counting criteria were applied consistently throughout the analysis. Values were expressed graphically as the mean per group ± SEM.

### 4.4. Stereological Analysis

To evaluate the neuronal density in the CA1 region, we modified the method used by [41]. The slides were first deparaffinized and subsequently rehydrated with distilled water as outlined above in the Immunofluorescence Section. The next step was Nissl staining with cresyl violet (0.1%), for which the slides were immersed in a cresyl violet solution for 5 min. This was followed by a dehydration phase using alcohol at increasing concentrations (80, 96, 100, and 100%) for one minute each. They were then immersed in xylene for 5 min. Finally, the slides were mounted with synthetic resin and the coverslip was applied. They were then allowed to air dry.

Neurons were sampled by optical sectioning within a volume in the CA1 region of the hippocampus, using an Olympus BH2 microscope with a color digital camera connected to a DataCell computer-assisted image analysis system (Stereo Investigator System; MicroBrightField, Williston, VT, USA) for stereology. From each case, at least four random sections were analyzed. The region of interest was outlined in a 600 × 600 μm contour using a systematic random sampling procedure. A 70 × 70 × 25 μm counting frame, with guard zones of 2.5 μm, was used for XYZ scanning through a UPlanSAPO water immersion objective at 60×. Counting criteria included cells within the counting frame or in contact with the inclusion boundaries, while excluding those in contact with the exclusion boundaries or outside the dissector. The identification of pyramidal neurons in the CA1 layer was based on anatomical and cytological criteria observable under a microscope. These criteria included morphology such as a pyramidal soma, relatively large cell size, a border and cytoplasmic appearance surrounding the nucleus, and a discernible stained nucleolus [61]. Results were expressed as the total number of neurons per mm3 and averaged by group. The precision of stereological estimates was assessed using the Gundersen Coefficient of error (CE, m = 1), which provides an estimate of the sampling variance associated with the stereological procedure. Lower CE values indicate greater precision of the cell count estimates [62]. The CE values obtained for each experimental group are presented in Table 1. The stereology data are detailed in the Supplementary Material.

### 4.5. Statistical Analysis

The absence of aberrant data was verified, and the normality of the data distribution was assessed using the Shapiro–Wilk test. Data were analyzed using a two-way ANOVA, with age and suicide status as independent variables, followed by Bonferroni’s post hoc test for multiple comparisons. Statistical significance was set at *p* < 0.05. Additionally, effect sizes (η^2^) were calculated, with values ≥ 0.14 considered indicative of a large effect. All analyses were performed using GraphPad Prism 9 (GraphPad Software, San Diego, CA, USA).

## 5. Conclusions

Immunodetection of glial, mitochondrial, and glucocorticoid receptor markers in the CA1 region of the hippocampus was decreased in individuals who died by suicide, regardless of age group. Notably, pyramidal cell density was decreased in young individuals who died by suicide, whereas no significant difference was observed in adults. These findings suggest age-related differences in hippocampal cellular alterations associated with suicide. Further studies are needed to determine whether these changes are associated with neuroinflammatory processes, altered cellular metabolism, or HPA axis dysregulation, and to elucidate their potential contribution to the neurobiology of suicide.

## Figures and Tables

**Figure 1:**
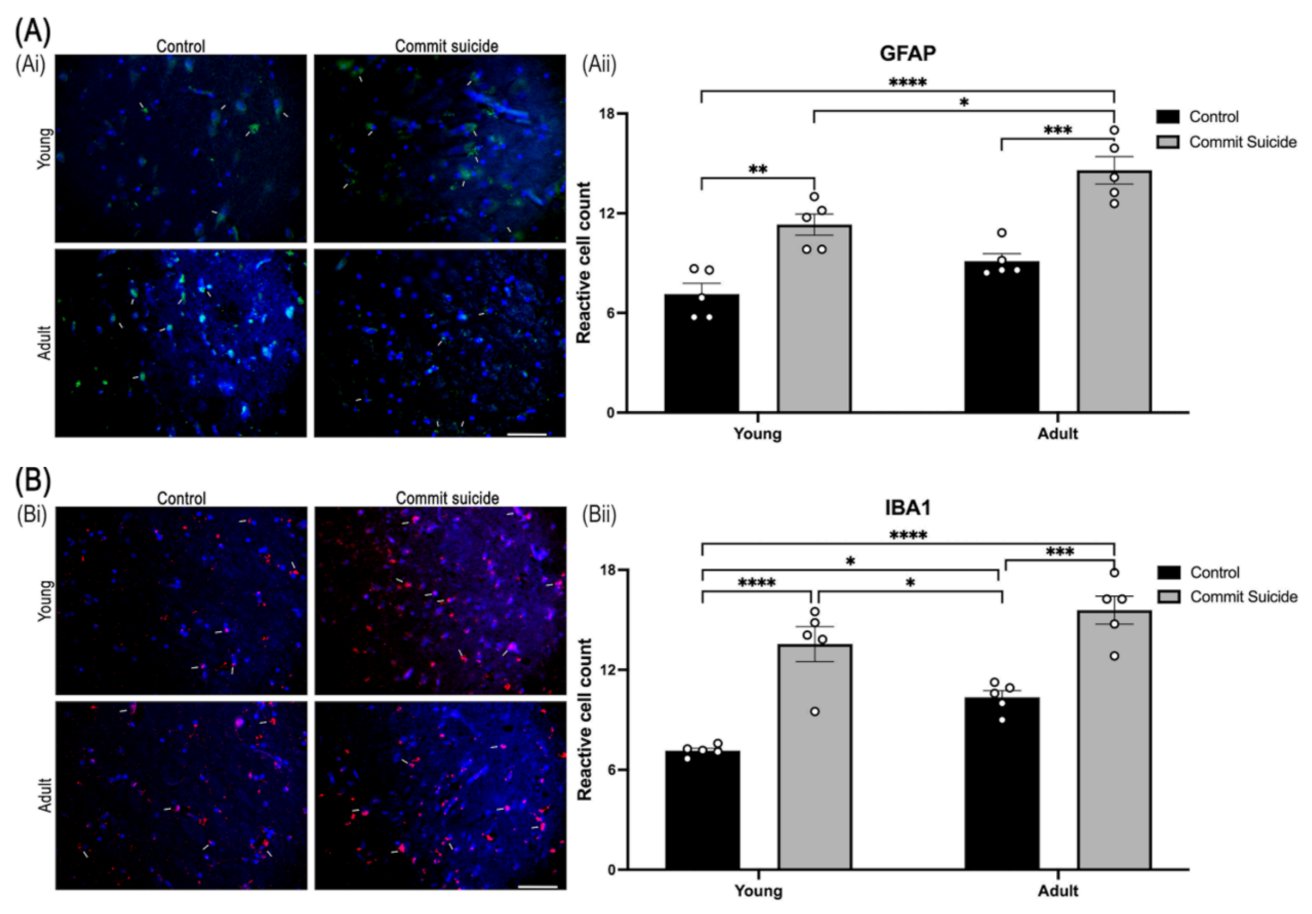
**GFAP and Iba1 immunoreactivity in the hippocampal CA1 region.** (**A**) GFAP immunoreactivity. (**Ai**) Representative photomicrographs of GFAP immunostaining for each experimental group. Scale bar = 50 µm. (**Aii**) Quantification of GFAP-positive cells in the CA1 region. Increased GFAP immunoreactivity was observed in both suicide groups compared with their respective controls. (**B**) Iba1 immunoreactivity. (**Bi**) Representative photomicrographs of Iba1 immunostaining for each experimental group. (**Bii**) Quantification of Iba1-positive cells in the CA1 region. Increased Iba1 immunoreactivity was observed in both suicide groups compared with their respective controls (two-way ANOVA; * *p* < 0.05, ** *p* < 0.01, *** *p* < 0.001, **** *p* < 0.0001).

**Figure 2:**
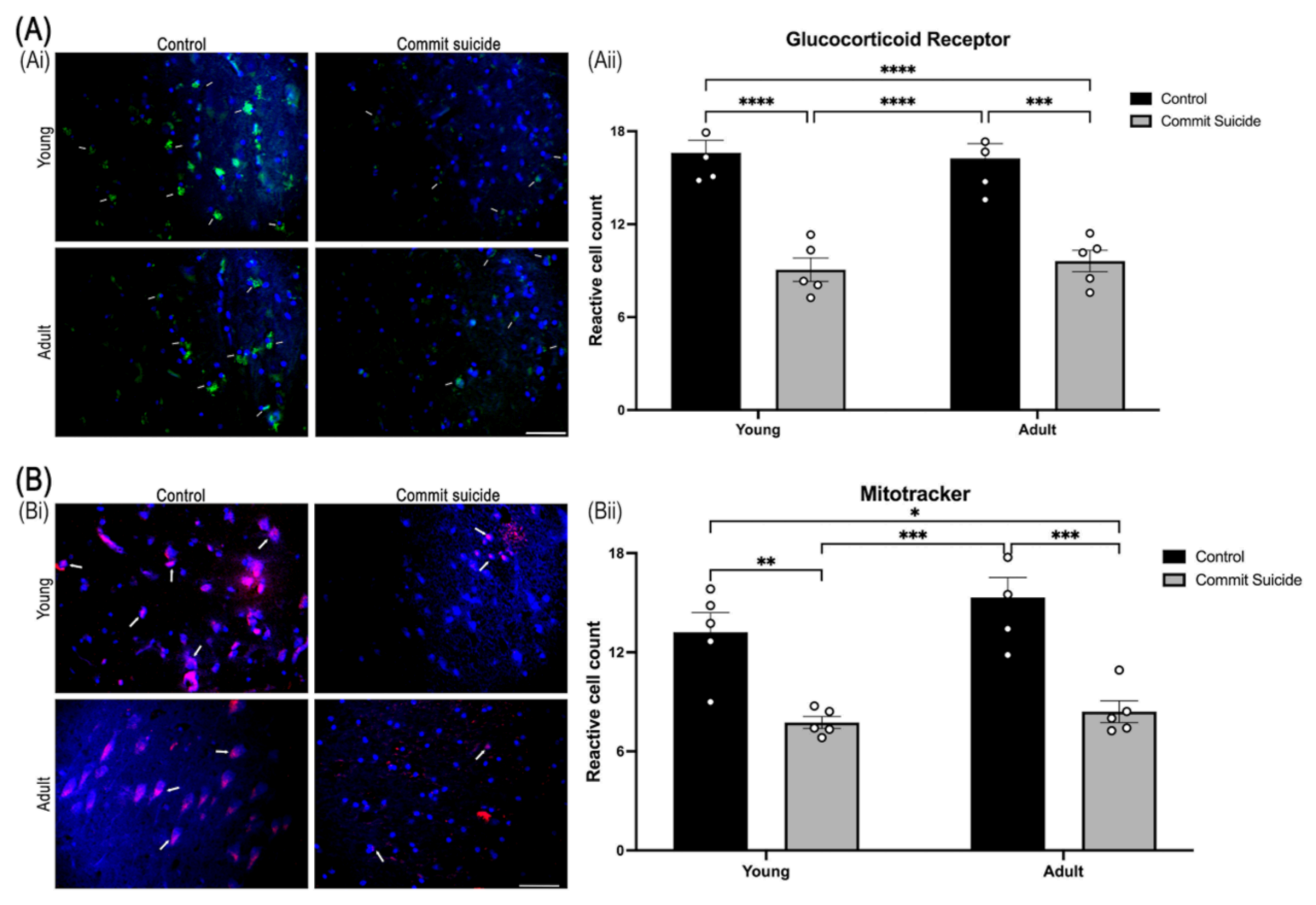
**Glucocorticoid receptor immunoreactivity and MitoTracker labeling in the hippocampal CA1 region.** (**A**) Glucocorticoid receptor immunoreactivity. (**Ai**) Representative photomicrographs of glucocorticoid receptor immunostaining for each experimental group. GFAP immunoreactivity is shown in green, and cell nuclei are counterstained with DAPI (blue). Scale bar = 50 µm. (**Aii**) Quantification of glucocorticoid receptor-positive cells in the CA1 region. Reduced glucocorticoid receptor immunoreactivity was observed in both suicide groups compared with their respective controls. (**B**) MitoTracker™ Red CMXRos was used for mitochondrial labeling (for details, see the Section 4.2). (**Bi**) Representative photomicrographs of MitoTracker™ labeling for each experimental group. Iba1 immunoreactivity is shown in red, and cell nuclei are counterstained with DAPI (blue). (**Bii**) Quantification of MitoTracker™-positive cells in the CA1 region. Both suicide groups exhibited a reduced number of MitoTracker™-positive cells compared with their respective controls (two-way ANOVA; * *p* < 0.05, ** *p* < 0.01, *** *p* < 0.001, **** *p* < 0.0001).

**Figure 3:**
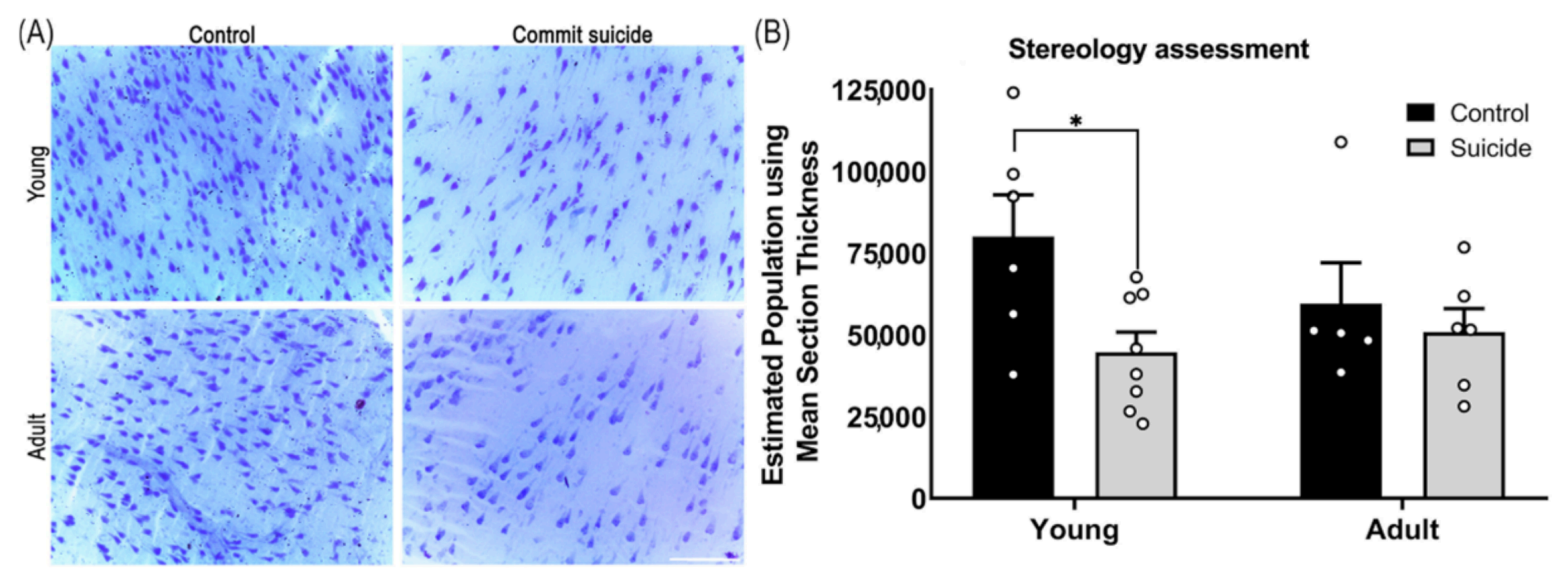
**Neuronal density in the hippocampal CA1 region assessed by stereological analysis.** (**A**) Representative photomicrographs of cresyl violet-stained sections from each experimental group. Cresyl violet staining appears violet to blue-violet, highlighting neuronal cell bodies and Nissl substance. Scale bar = 100 µm. (**B**) Quantification of neuronal density in the CA1 region obtained using stereological methods. No significant differences were observed between the Adult Control and Adult Suicide groups. In contrast, the Young Suicide group exhibited a significant reduction in neuronal density compared with the Young Control group (two-way ANOVA; * *p* < 0.05).

**Figure 4:**
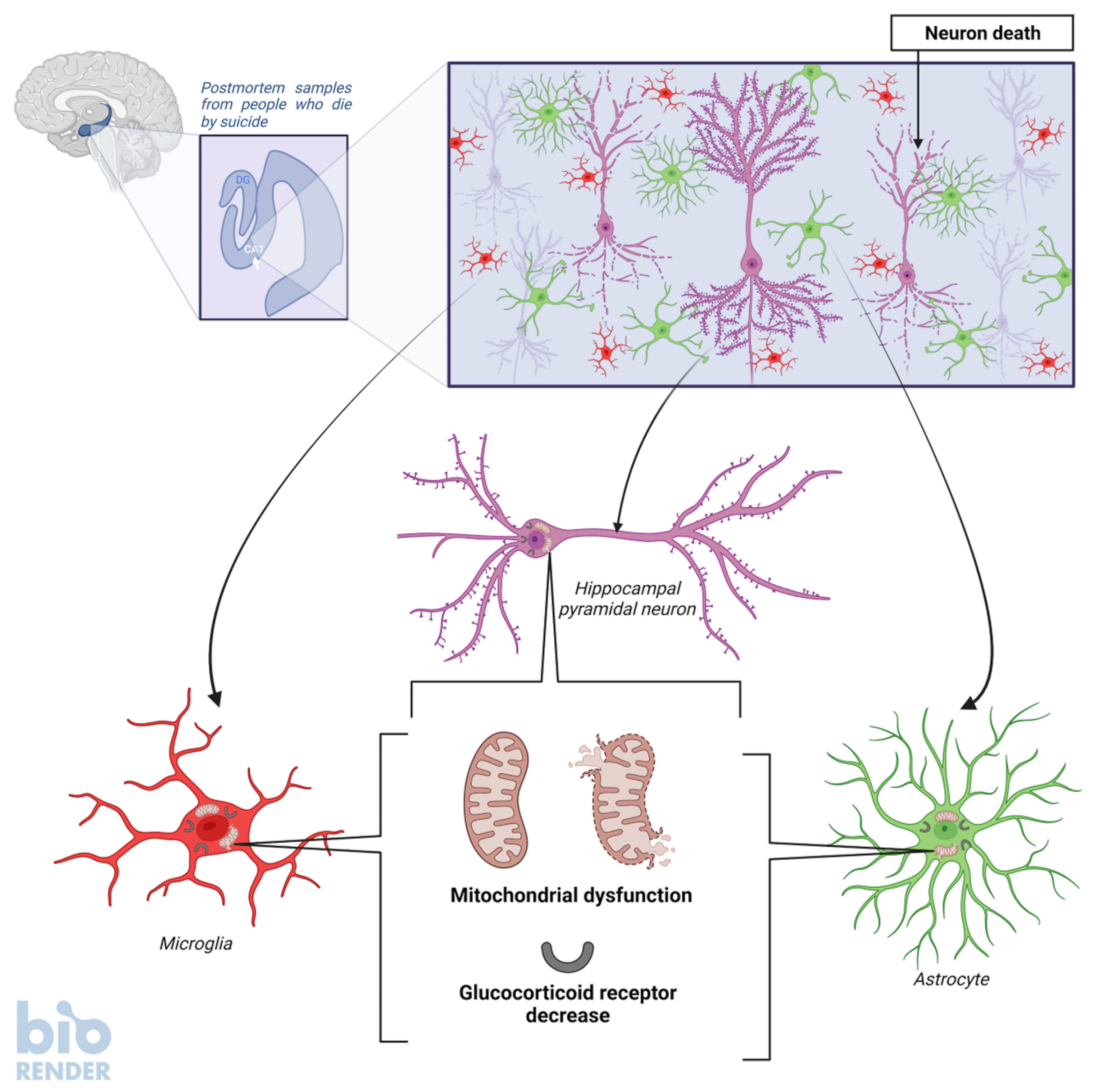
**Hypothetical mechanism.** The model integrates the reduced neuronal density observed in pyramidal neurons, increased numbers of Iba1-positive microglia and GFAP-positive astrocytes, decreased glucocorticoid receptor immunoreactivity, and reduced MitoTracker™ labeling. Together, these alterations may reflect interconnected changes in glial reactivity, stress-response signaling, and mitochondrial density that contribute to hippocampal dysfunction associated with suicide. The diagram highlights changes in hippocampal pyramidal neurons (purple), microglia (red), and astrocytes (green); arrows point to the cells. This schematic represents a hypothetical interpretation of the present findings and does not imply direct causal relationships. Created in BioRender. Vazquez, A. (2026) https://BioRender.com/9zei4aa (accessed on 25 October 2026).

**Figure 5:**
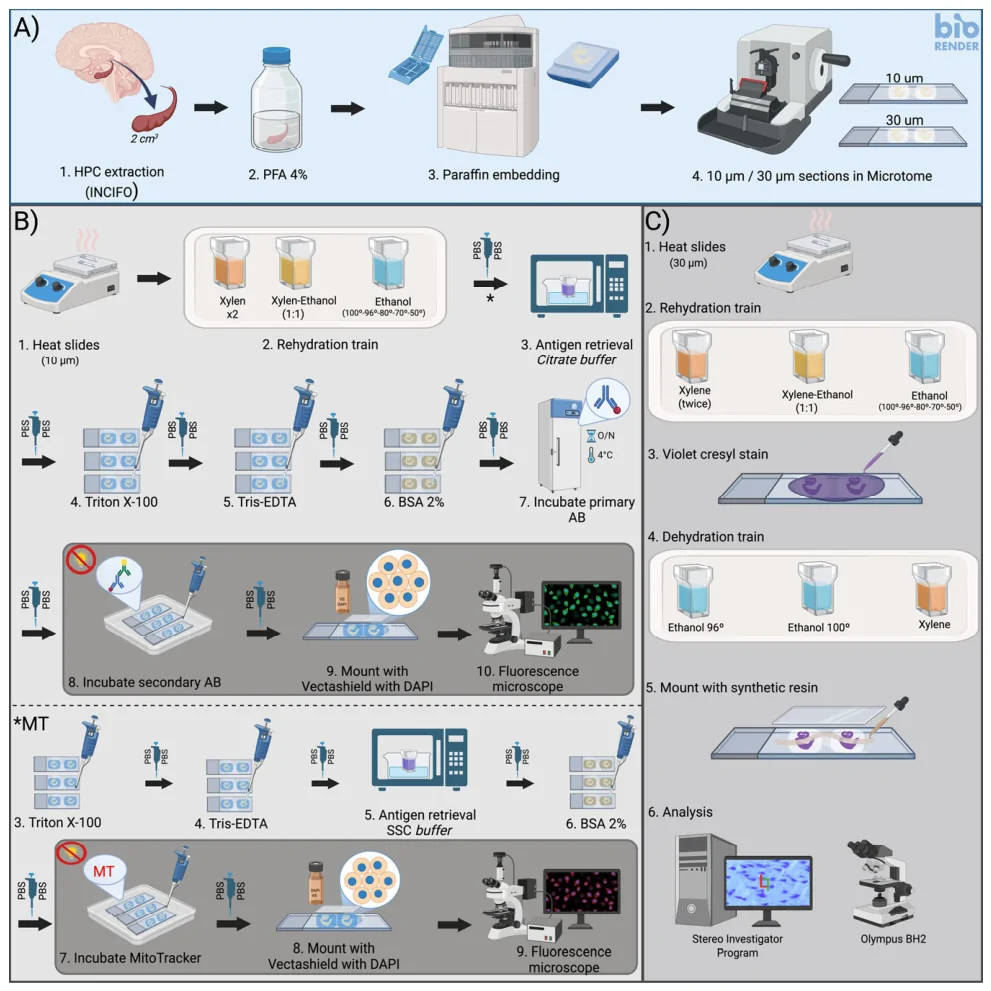
**Schematic representation of the experimental protocol.** (**A**) Hippocampal (HPC) samples were obtained from INCIFO, processed, and sectioned. (**B**) Immunohistochemistry workflow; arrows indicate the sequence of procedures. After the second step, the asterisk (*) denotes the mitochondrial labeling assay (for details, see the Section 4.2). (**C**) Stereological analysis protocol. Abbreviations: AB, antibody; BSA, bovine serum albumin; MT, MitoTracker; O/N, overnight; PBS, Phosphate Saline Buffer; PFA, paraformaldehyde; SSC, Saline Sodium Citrate. Created in BioRender. Vazquez, A. (2026) https://BioRender.com/qlqvlqj (accessed on 25 October 2026).

**Table 1:** Demographic and clinical characteristics of the cases included in the study.

GROUP	HEMISPHERE	AGE	CAUSE OF DEATH	ETIOLOGY	PSYCHIATRIC DIAGNOSIS	GUNDERSEN COEFFICIENT m = 1 (<0.1)	Essays
**Young Suicide**	Left	21–25	Asphyxia due to hanging	Undetermined	Undetermined	0.06	IF/St
**Young Suicide**	Right	21–25	Asphyxia due to hanging	Relationship problems	No	0.09	IF/St
**Young Suicide**	Right	21–25	Asphyxia due to hanging	Undetermined	Undetermined	0.08	IF/St
**Young Suicide**	Right	16–20	Asphyxia due to hanging	Undetermined	Undetermined	0.09	St
**Young Suicide**	Left	21–25	Asphyxia due to hanging	Undetermined	Undetermined	0.09	St
**Young Suicide**	Right	21–25	Asphyxia due to hanging	Depression	Yes	0.05	IF/St
**Young Suicide**	Right	16–20	Asphyxia due to hanging	Undetermined	Undetermined	0.05	IF/St
**Young Suicide**	Left	26–30	Asphyxia due to hanging	Undetermined	Undetermined	0.06	St
**Adult Suicide**	Right	56–60	Asphyxia due to hanging	Undetermined	Undetermined	0.06	IF/St
**Adult Suicide**	Right	61–65	Asphyxia due to hanging	Depression	Yes	0.08	IF/St
**Adult Suicide**	Left	41–45	Asphyxia due to hanging	Depression	Yes	0.07	St
**Adult Suicide**	Right	31–35	Asphyxia due to hanging	Undetermined	Undetermined	0.08	IF/St
Adult Suicide *	Left	51–55	Asphyxia due to hanging	Depression	Yes	0.15*	IF/St
**Adult Suicide**	Left	41–45	Asphyxia due to hanging	Depression	Yes	0.08	St
**Adult Suicide**	Right	31–35	Asphyxia due to hanging	Depression	Yes	0.07	IF/St
**Young Control**	Right	21–25	Gunshot wound	Cranio-thoracic gunshot wound	Undetermined	0.07	St
**Young Control**	Left	21–25	Septic shock	Septic shock	Undetermined	0.07	IF/St
**Young Control**	Right	21–25	Asphyxia due to strangulation	Asphyxia due to strangulation	Undetermined	0.06	IF/St
**Young Control**	Right	16–20	Craniocerebral trauma (traumatic brain injury)	Fatal injuries sustained in a traffic collision	Undetermined	0.06	IF/St
**Young Control**	Left	16–20	Cardiopulmonary infarction	Pulmonary edema	Undetermined	0.07	IF/St
**Young Control**	Left	16–20	Sharp-force injury	Thoracic penetrating injury	Undetermined	0.07	IF/St
**Adult Control**	Left	41–45	Acute myocardial infarction	Acute myocardial infarction	Undetermined	0.07	IF/St
**Adult Control**	Left	31–35	Asphyxia due to strangulation	Asphyxia	Undetermined	0.06	IF/St
**Adult Control**	Left	41–45	Sharp-force injury	Machete-inflicted neck wound	Undetermined	0.07	IF/St
**Adult Control**	Left	41–45	Multiple traumatic injuries	Multiple traumatic injuries	Undetermined	0.09	IF/St
**Adult Control**	Left	31–35	Gunshot wound	Gunshot wound to the thorax	Undetermined	0.07	IF/St

Abbreviations: IF, immunofluorescence; St, stereological cell count. * This case was excluded from the stereological analysis due to its Gundersen Coefficient value.

## Data Availability

The raw data supporting the conclusions of this article will be made available by the authors on request.

## Supplementary Materials

The following supporting information can be downloaded at https://www.mdpi.com/article/10.3390/ijms27219700/s1.

## Abbreviations

The following abbreviations are used in this manuscript:

PFC	Prefrontal cortex
NAcc	Nucleus accumbens
BLA	Basolateral amygdala
DM	Dorsomedial
HPA	Hypothalamic–pituitary–adrenal
CA	Corpus ammonis
MHCII	Major histocompatibility complex, class II
GFAP	Glial fibrillary acidic protein
Iba1	Ionized calcium-binding adapter molecule 1
INCIFO	Institute of Forensic Sciences
PBS	Phosphate-buffered saline
BSA	Bovine serum albumin
ACTH	Adrenocorticotropic hormone
CRH	Corticotropin-releasing hormone
