# Supplementary material for: Hippocampal Glial Alterations Are Associated with Mitochondrial Changes and Reduced Glucocorticoid Receptor Density in Suicides
Source: Int J Mol Sci. 2026 Oct 29;27(21):9700. doi: 10.3390/ijms27219700 (PMC13763202; doi:10.3390/ijms27219700)
Supplement: Supplementary file 1 [file ijms-27-09700-s001.zip › ijms-4629436-supplementary.pdf]

Supplementary Material

Case selection and inclusion/exclusion criteria.

All postmortem hippocampal tissue samples were obtained from the Tissue collection of the Institute of Forensic Science (INCIFO), following approval by the corresponding institutional ethics committee. Because well-characterized hippocampal tissue from the human brain is limited, the present study was based on the cases available in the institutional collection at the time of sample acquisition.

Case classification was established by a board-certified forensic pathologist, following the complete medico-legal investigation, including the official determination of the manner of death. Cases included in the present study met the following inclusion criteria:

- Availability of hippocampal tissue containing a well-preserved CA1 region.
- Adequate tissue preservation.
- Complete demographic information (age, sex, psychiatric history, toxicological findings, and tissue laterality, see table 1).
- Availability of cause and manner of death established by forensic investigation.
- Ages for young group (18-29 years old) and adult group (31-76 years old).

For the cases of suicide, the following was considered: the forensic authority's classification of the death as suicide after a complete medico-legal investigation. For the control cases, the following was considered: no history or forensic evidence of suicide.

Exclusion criteria for all groups included:

- Samples showing inadequate tissue preservation.
- Insufficient material for the planned analyses.
- Samples with inability to reliably identify the CA1 region.
- Ages higher or lower than those established in inclusion criteria.

Stereology data

All neuronal estimates were obtained using the Optical Fractionator probe in Stereo

GROUP	GUNDERSEN COEFICIENT m=1	Sampling sites (n)	Mean mounted thickness (μm)	Number-weighted thickness (μm)	Total neurons counted
**Young suicide**	0.06	81	37.0	38.2	282
**Young suicide**	0.09	57	53.1	55.6	122
**Young suicide**	0.08	83	32.0	32.4	174
**Young suicide**	0.09	48	34.1	35.2	114
**Young suicide**	0.09	62	33.1	33.8	137
**Young suicide**	0.05	79	61.2	61.5	466
**Young suicide**	0.05	89	64.6	65.2	428
**Adult suicide**	0.06	94	36.1	35.5	362
**Adult suicide**	0.08	67	31.0	31.3	190
**Adult suicide**	0.07	82	35.1	35.4	250
**Adult suicide**	0.08	80	30.3	30.3	158
**Adult suicide**	0.15	16	32.0	34.1	56
**Adult suicide**	0.07	63	38.8	38.7	201
**Young control**	0.07	63	33.1	32.9	195
**Young control**	0.07	48	46.1	45.5	260
**Young control**	0.06	54	59.2	59.5	285
**Young control**	0.06	85	30.4	30.3	316
**Young control**	0.07	48	62.7	62.3	337
**Young control**	0.07	63	60.9	61.0	258
**Adult control**	0.07	60	39.0	44.3	224
**Adult control**	0.06	84	29.8	29.9	276
**Adult control**	0.08	56	58.8	58.9	179
**Adult control**	0.09	44	67.2	68.4	136
**Adult control**	0.07	70	30.1	30.1	218
**Adult control**	0.09	56	71.7	72.0	120
**Adult control**	0.07	60	60.8	60.9	305

Investigator (MBF Bioscience). The same stereological sampling parameters were

applied to all cases: counting frame = 70 × 70 μm (4,900 μm²), sampling grid = 600 × 600 μm, optical dissector height = 25 μm, guard zone = 2.5 μm, number of sections analyzed = 8, and section evaluation interval = 2.

Section thickness was measured directly at the stereological sampling sites using Stereo Investigator, and population estimates were calculated using the measured section thickness rather than the nominal section thickness. As shown in the table, considerable inter-case variability in measured post-processing thickness was observed. Because the original sections are no longer available for independent remeasurement, these values are reported transparently, and the observed variability should be considered when interpreting the stereological estimates.
